# Supplementary material for: Universality and quantum criticality in quasiperiodic spin chains
Source: Nat Commun. 2020 May 6;11:2225. doi: 10.1038/s41467-020-15760-5 (PMC7203221; doi:10.1038/s41467-020-15760-5)
Supplement: Supplementary file 1 — Supplementary Information [file 41467_2020_15760_MOESM1_ESM.pdf]

Universality and Quantum Criticality in Quasiperiodic Spin Chains

Agrawal et al.

SUPPLEMENTARY DISCUSSION

Supplementary Note 1.

This section elaborates on various aspects of the ground-state physics of quasiperiodic spin chains. The key result of this section is an explanation of why generic quasiperiodic patterns flow to sequences; we also discuss other aspects of quantum criticality in these spin chains.

A. Pattern of the local minima in the initial potential

We noted in the main text that the local minima of the initial potential $1 + \cos(2\pi\varphi i + \theta)$—i.e., sites at which ℓ is smaller than at either of the neighbors—follow a sequence structure that sharpens into a discrete sequence under renormalization. We here provide a proof of this statement for this specific potential. It is helpful, for this section, to define the *fractional part* of a real number x, denoted $\{x\}$, as the difference between x and the largest integer less than x, i.e., $\{x\} \equiv x - \lfloor x \rfloor$.

We start by analyzing the pattern of minima in the initial potential. Let us suppose that for a given coupling n, $\ell_n = 1 + \cos 2\pi\varphi n$ is a local minimum (we take $\theta = 0$ for now). By definition, we have $\ell_{n+1} > \ell_n$. Let $y \equiv (n+1)\varphi$ and $x \equiv n\varphi$. There are two cases: $\{x\} < 1/2$ and $\{x\} > 1/2$. When $\{x\} < 1/2$, $\ell_{n+1} > \ell_n$ implies that $\{y\} \in (0, \{x\}) \cup (1 - \{x\}, 1)$, whereas if $\{x\} > 1/2$ then $\{y\} \in (0, 1 - \{x\}) \cup (\{x\}, 1)$.

Studying various cases with $0 < \{n\varphi\} < 1 - \{\varphi\}$, $1 - \{\varphi\} < \{n\varphi\} < 1/2$ and $\{n\varphi\} > 1/2$, we get the condition for $\ell_{n+1} > \ell_n$ to be $\frac{1-\{\varphi\}}{2} < \{n\varphi\} < 1 - \frac{\{\varphi\}}{2}$. Applying the same procedure to the inequality $\ell_{n-1} > \ell_n$, we find $\frac{\{\varphi\}}{2} < \{n\varphi\} < \frac{1+\{\varphi\}}{2}$. Taking the intersection of these two inequalities, the condition for ℓ_n to be a local minimum reads

$$\frac{\{\varphi\}}{2} < \{n\varphi\} < 1 - \frac{\{\varphi\}}{2}.$$

Reintroducing the phase θ to the initial potential modifies the above result to $\frac{\{\varphi\}}{2} < \{n\varphi + \frac{\theta}{2\pi}\} < 1 - \frac{\{\varphi\}}{2}$. This can be written as,

$$\boxed{0 < \{n\varphi + \frac{\theta}{2\pi} - \frac{\{\varphi\}}{2}\} < 1 - \{\varphi\}.}$$

We now identify this pattern with the Fibonacci binary sequence defined by the inflation rules $A \to AB$, $B \to A$. Recall that the inflation rule is equivalent to stating that the site n is a B site iff $\lfloor (n + n_0 + 1)\varphi \rfloor - \lfloor (n + n_0)\varphi \rfloor = 1$, where the sequence—starting from the initial letter A, then applying the inflation rules—begins at site n_0. Here, $\lfloor \cdot \rfloor$ denotes the largest integer smaller than $\cdot$. Thus, n_0 merely translates the standard Fibonacci sequence. Writing $\lfloor (n + n_0 + 1)\varphi \rfloor$ as $\lfloor (n + n_0)\varphi \rfloor + \lfloor \varphi \rfloor + \lfloor \{(n + n_0)\varphi\} + \{\varphi\} \rfloor$ yields $\lfloor \{(n + n_0)\varphi\} + \{\varphi\} \rfloor = 0$. This is true if and only if $0 < \{n\varphi + n_0\varphi\} < 1 - \{\varphi\}$.

Thus we have the following chain of results:

$$(\ell_n \text{ is a local minimum}) \iff 0 < \left\{ n\varphi + \frac{\theta}{2\pi} - \frac{\{\varphi\}}{2} \right\} < 1 - \{\varphi\} \iff$$

$$\Big(\text{letter `}B\text{' is at } n^{\text{th}} \text{ position in the Fibonacci word sequence shifted from the standard Fibonacci sequence by distance } n_0 \text{ such that } \{n_0\varphi\} = \{\frac{\theta}{2\pi} - \frac{\{\varphi\}}{2}\}\Big),$$

proving the claim that local minima of the initial potential indeed follow the pattern of the letter 'B' in the Fibonacci sequence.

Thanks to the above result, we can decompose the cosine coupling into A-couplings and B-couplings arranged in a Fibonacci sequence. To emphasize this decomposition, we shall relabel the couplings ℓ_n as follows:

$$\ell_n \equiv \begin{cases} A_0(n), & \ell_n \text{ is not a minimum} \\ B_0(n), & \ell_n \text{ is a minimum} \end{cases} \tag{1}$$

Note that $A_0(n)$ and $B_0(n)$ are not defined for all values of n: for a given value of n, either $A_0(n)$ or $B_0(n)$ is defined.

B. Sharpening of the sequence structure under RG

For the XXX spin chain, the RG rules are given by

$$\ell_{\text{eff}} = \ell_{n+1} - \ell_n + \ell_{n-1} + c, \tag{2}$$

with $c = \ln 2$ where ℓ_n is the smallest ℓ. We remark that the whole Fibonacci sequence is built up of word patterns 'ABABA' and 'ABA'. This means that we can apply the decimate all the local minima B in one go, arriving at the following renormalized couplings:

$$\begin{aligned} A_{m+1}(n) &= A_m(n-2) - B_m(n-1) + A_m(n) - B_m(n+1) + A_m(n+2) + 2c \\ B_{m+1}(n) &= A_m(n-1) - B_m(n) + A_m(n+1) + c, \end{aligned}$$

where m labels the number of such Fibonacci RG steps. In order to get A_{m+1} we decimate two B_m's, and to get B_{m+1} we decimate one B_m. If A_m and B_m form a Fibonacci sequence then A_{m+1} and B_{m+1} also follow the Fibonacci pattern. This follows from the inflation rule: $A \to ABABA$ and $B \to ABA$. Thus starting from a Fibonacci word sequence, if we replace 'ABABA' by 'A' and 'ABA' by 'B', we again get a new Fibonacci sequence.

Note: We are considering periodic boundary conditions, so our initial system size should be an even number. Also we will consider system sizes given by Fibonacci numbers, F_n, as the fractional part of $F_n\varphi$ is φ^{-n}, *i.e.* $F_n\varphi \approx F_{n+1}$. Since all even Fibonacci numbers can be written as F_{3l}, we consider initial system sizes of the form $N = F_{3l}$. In a Fibonacci sequence of size F_{3l}, we have F_{3l-2} B letters and F_{3l-1} A letters. Since this is a word sequence we can decimate all the F_{3l-2} 'B' couplings in one go. Each decimation decreases the number of couplings (bonds) by 2. So after F_{3l-2} decimations, the number of remaining bonds is $F_{3l} - 2F_{3l-2} = F_{3(l-1)}$. This corresponds to what we call a Fibonacci step.

After the first Fibonacci step, we have two bands of couplings, A and B, given by,

$$\begin{aligned} A_1(n) &= \sum_{i=-2,0,2} (1+\cos(2\pi\varphi(\mathrm{n}+\mathrm{i}))) - \sum_{i=-1,1} (1+\cos(1+2\pi\varphi(\mathrm{n}+\mathrm{i}))) + 2\mathrm{c}, \\ &= 1 + 2c + \cos(2\pi\varphi\mathrm{n})(1 + 2(\cos(4\pi\varphi) - \cos(2\pi\varphi))), \\ &= 1 + 2c + \lambda_1 \cos(2\pi\varphi\mathrm{n}). \\ B_1(n) &= \sum_{i=-1,1} (1+\cos(1+2\pi\varphi(\mathrm{n}+\mathrm{i}))) - (1+\cos(2\pi\varphi(\mathrm{n})) + \mathrm{c}, \\ &= 1 + c - \cos(2\pi\varphi\mathrm{n})(1 - 2\cos(2\pi\varphi)), \\ &= 1 + c + \lambda_2 \cos(2\pi\varphi\mathrm{n}), \end{aligned}$$

where $(1 + 2(\cos(4\pi\varphi) - \cos(2\pi\varphi)) \equiv \lambda_1 \approx 2.64$ and $-(1 - 2\cos(2\pi\varphi)) \equiv \lambda_2 \approx -2.47$. The label n in $A_1(n)$ and $B_1(n)$ only takes certain integer values, corresponding respectively to the positions—on the original lattice—of the central bonds on the $ABABA$ and ABA clusters. Thus for instance (Fig. 1) if $A_1(0)$ is defined, at the center of the A cluster, then $B_1(n)$ can only be defined for $n = 4$. One can straightforwardly check that at this level all $B_1(n)$ that are defined are local minima, i.e., they are smaller than the neighboring A_1 couplings.

By considering the range of possible values of n [i.e., the on-site phase] for which an ABABA sequence is possible, we find the following constraint:

$$1 - \cos(2\pi\varphi n) < \delta A_1, \quad \delta A_1 = 1 - \cos\left(2\pi\frac{-3\varphi+5}{2}\right) [\approx 0.1]. \tag{3}$$

Thus the bandwidth for A-type couplings on the new, decimated chain is ≈ 0.1. A similar analysis for the B couplings—now considering patterns of the form AABAA—yields the constraint $\cos(2\pi\varphi n) \in (-1, -1 + \delta \mathrm{B}_1)$ where $\delta B_1 = 1 - \cos\left(2\pi\frac{5\varphi-8}{2}\right)$ (≈ 0.04). See Fig. 1. To summarize, after one step of decimation, all the couplings within each "type" (A or B) become similar in value, but are separated from couplings of the other type by $\approx c$. This leads to the formation of well-separated bands, which then become increasingly well-separated under the action of the RG.

To form A and B couplings in the subsequent Fibonacci steps, the restriction on the initial value of the middle couplings becomes tighter. For example, to get an A_2 coupling we need to have 'ABABA*ABA*ABABA*ABA*ABABA'

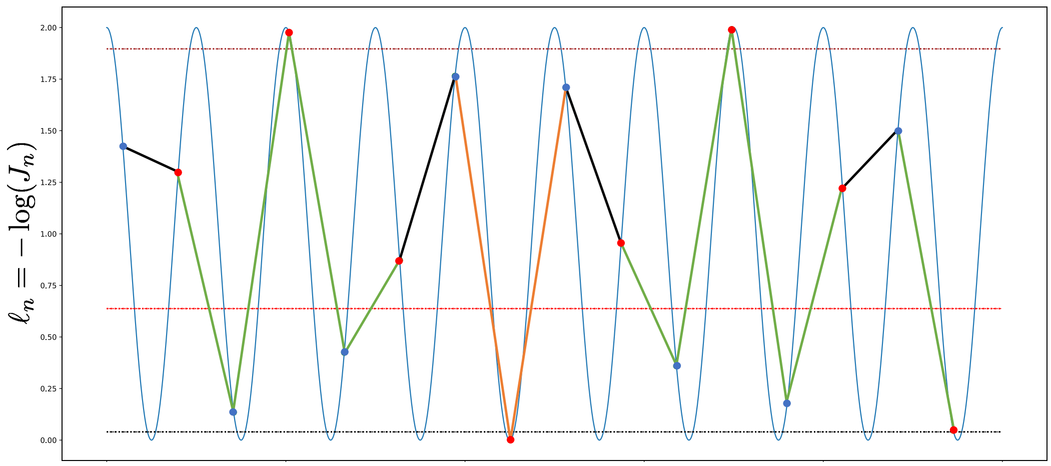

Supplementary Figure 1. Part of the initial coupling distribution. The green 'W' shape is the pattern 'ABABA' in the Fibonacci sequence; this will give rise to A block in the next generation of Fibonacci sequence. The orange 'V' shape is the pattern 'ABA' which will lead to a B block in the next generation. Any block with length in the region above the brown dashed line will be the middle 'A' block in the pattern 'ABABA', while blocks in the region between the red and black dashed line will constitute the 'B' blocks in 'ABABA'. Blocks with length below the blue dashed line will be a 'B' block in the pattern 'ABA'.

patterns in the initial potential, but to have such a long pattern the middle 'A' coupling needs to be closer to $\cos 2\pi\varphi n = 1$ than it was required to form A_1. Thus we expect the fluctuations δA_m and δB_m to go to zero as $m \to \infty$. Empirically these fluctuations decay exponentially with m.

This can be iterated to get (we are using $C(n) \equiv \cos 2\pi\varphi n$ and $S(n) \equiv \sin 2\pi\varphi n$ for brevity),

$$\begin{aligned}
A_m(n) &= \sum_{i=-\frac{F_{3m+2}-1}{2}}^{\frac{F_{3m+2}-1}{2}} (-1)^i \left(1 + \cos 2\pi\varphi(n+i)\right) + m(m+1)c, \\
&= 1 + m(m+1)c \ + C(n)\left(1 + 2\frac{S(\frac{F_{3m+2}-1}{4})}{S(1)}\left(C\left(\frac{F_{3m+2}+3}{4}\right) - C\left(\frac{F_{3m+2}-1}{4}\right)\right)\right), \\
&= 1 + m(m+1)c + C(n)\left(1 - 2\frac{S(\frac{F_{3m+2}-1}{4})S(\frac{F_{3m+2}+1}{4})}{C(1/2)}\right), \\
&= 1 + m(m+1)c + C(n)\left(1 - \frac{C(1/2) - C(\frac{F_{3m+2}}{2})}{C(1/2)}\right), \\
&= 1 + m(m+1)c + \frac{C(\frac{F_{3m+2}}{2})}{C(1/2)}C(n).
\end{aligned}$$

Similarly we have

$$\begin{aligned}
B_m(n) &= 1 + m^2c - (-1)^m C(n)\frac{C(\frac{F_{3m+1}-3}{4\varphi} + \frac{F_{3m+1}}{2})}{C(1/2)}, \\
&= 1 + m^2c - (-1)^m C(n)\frac{(-1)^{m+1}C(\frac{F_{3m+1}}{2})}{C(1/2)}, \\
&= 1 + m^2c + \frac{C(\frac{F_{3m+1}}{2})}{C(1/2)}C(n).
\end{aligned}$$

For large m, $A_m(n) \approx 1 + m(m+1)c + \frac{1}{\cos\pi\varphi}$ and $B_m(n) \approx 1 + m^2c + \frac{1}{\cos\pi\varphi}$. $A_m - B_m \xrightarrow{m\to\infty} mc$. If $c = 0$ (corresponding physically to Ising and XX chains that can be mapped onto free fermions), then the sequence structure

does not survive under RG.

C. Generic potentials

We showed above that for couplings of the form $-\log(J_n) = a + \cos(2\pi\varphi n + \theta)$, the initial distribution flows to a sequence under RG. But this result is quite general and almost all initial distribution of the couplings flow to a sequence. If f is any monotonic function in the range $(-1, 1)$, is bounded and $\ell_n = -\log J_n = f(\cos(2\pi\varphi n + \theta))$, then the minima can be identified exactly as in the analysis above. Therefore, under RG, the couplings flow to the Fibonacci sequence. Natural functions like logarithm, exponential, etc. all satisfy the above criteria. For example, couplings of the form $\ell_n = -\log(1+\epsilon+\cos(2\pi\varphi n+\theta))$ (corresponding to the natural choice $J_n = 1+\epsilon+\cos(2\pi\varphi n+\theta)$) will flow to a sequence – and we verified this numerically – where ϵ is a positive constant which prevents the couplings from hitting the singularity. We have also checked that adding higher harmonics to J_n such as $\cos(4\pi\varphi n + \delta)$ with a different phase δ does not affect the flow to sequences.

Note that f need not be a monotonic function. E.g for $f(x) = |0.5 + x|$ and $f(x) = \sin(2x)$, we get Fibonacci sequences under RG flow even though the above functions are non-monotonic in the range (-1,1). But if the function f has too many extrema, we find that the Fibonacci sequence structure gets destroyed, e.g $f(x) = \sin(4x)$.

D. Correlation length exponent

The fixed points of the above RG is unstable against dimerization of even and odd couplings. To find the critical exponent ν associated with the fixed point, we study the behavior of the sequence under an asymmetric perturbation. We dimerize the perfect sequence as $A_0^E = A_0 + \delta^E$, $B_0^E = B_0 + \delta^E$, $A_0^O = A_0 - \delta^O$, $B_0^O = B_0 - \delta^O$. Under RG, after m Fibonacci steps the sequence flows to,

$$A_m^{E,O} = (2m+1)A_0 - 2mB_0 + m(m+1)c \pm \frac{F_{3m+2}+1}{2}\delta^{E,O} \pm \frac{F_{3m+2}-1}{2}\delta^{O,E}$$
$$B_m^{E,O} = 2mA_0 - (2m-1)B_0 + m^2 c \pm \frac{F_{3m+1}+1}{2}\delta^{E,O} \pm \frac{F_{3m+1}-1}{2}\delta^{O,E}.$$

The asymmetry between even and odd blocks keeps increasing with m and eventually $B^E - A^O = B_0 - A_0 - mc + \frac{F_{3m+3}}{2}(\delta^E + \delta^O)$ will become positive, destroying the Fibonacci pattern and driving the system to a phase. Taking $\delta^E = \delta^O = \delta_0$, the dimerization parameter $\delta_m = A_m^E + B_m^E - (A_m^O + B_m^O) = F_{3m+3}\delta_0/2$ scales under RG as $\mathcal{R}[\delta_m] \equiv \delta_{m+1} \approx \varphi^3 \delta_m$ with the RG eigenvalue λ_δ given by $\lambda_\delta = \varphi^3 \equiv b^{y_\delta}$. In a single Fibonacci step, the system size is scaled by φ^3 implying that $b = \varphi^3$. This gives us $\nu \equiv 1/y_\delta = 1$.

E. Spin exponent for the quasiperiodic quantum Potts chain

At the critical point, the 2-point correlation function of the Potts order parameter scales as,

$$C(0, r) = \langle \sigma_0 \sigma_r \rangle \sim \frac{1}{r^{2\Delta_\sigma}}. \tag{4}$$

Assuming for now that the critical point of the quantum Potts model is given by the Fibonacci fixed point discussed above, we can calculate this 2-point function analytically. Decimating the J_i couplings in the RG leads to the formation of spin clusters which are locally ferromagnetic, while the decimations of a transverse field term h_i freezes the corresponding spin (or effective spin cluster) in a given configuration with zero magnetization. We call the spin clusters that are not yet decimated active. The 2-point function is equal to the probability of the spins at 0 and r to be part the same active cluster, which is true if and only if all the spins between them are either part of the same cluster to which the spins at 0 and r will eventually belong to, or are decimated. This implies that the probability for the two spins to be in same cluster is equal to the probability for them not to be decimated at the length scale where there is no other active cluster in between them. If we denote by $P(r)$ the probability for a given spin not be decimated under RG over a distance r, we thus have $C(r) \sim P(r)^2$.

We then compute $P(r) \sim r^{-\Delta_\sigma}$ as follows: we start with system size $r = F_{3m}$ with h_i and J_i taken from the same Fibonacci sequence, with h corresponding to odd links and J even ones. Let h_0 denote the magnetic field associated

with the cluster under consideration. For a Fibonacci sequence, we know that all 'B' couplings are decimated in a single Fibonacci step. Thus we have following relation,

$$\begin{aligned} P(r) &= (\text{Prob that } h_0 \text{ is not decimated}) \\ &= \prod_{i=0}^{m} P(h_0 \text{ is a 'A' type bond after } i \text{ Fibonacci steps}) \\ &\equiv \prod_{i=0}^{m} P_i. \end{aligned} \tag{5}$$

P_0 represents the probability that the relevant field h_0 is a type 'A' coupling in the initial distribution, and is given by $P_0 = \varphi/(1+\varphi)$. ($P_0$ is the probability of getting a 'A' coupling at some particular site for a randomly chosen global phase.) P_1 is the probability that the h_0 coupling is a type 'A' link after 1 Fibonacci step. P_1 thus denotes the probability that the bond in question is part of a sub-pattern in previous Fibonacci step which gives birth to a 'A' bond in this step. For the standard Fibonacci sequence we know this sub-pattern to be 'ABABA'. Thus P_1 = probability of the link to be in the sub-pattern 'ABABA' in the previous Fibonacci step $= 3\varphi/(3\varphi+2)$. In a similar fashion we can show that all remaining $P_i = 3\varphi/(3\varphi+2)$. This leads to,

$$P(r) = \frac{\varphi}{\varphi+1}\left(\frac{3\varphi}{3\varphi+2}\right)^{m-1} \sim \left(\frac{3\varphi}{3\varphi+2}\right)^{\ln r/3\ln\varphi} \sim r^{-\Delta_\sigma},$$

with $\Delta_\sigma = \ln\left(1+2\varphi^{-1}/3\right)/\left(3\ln\varphi\right)$.

For a quasiperiodic Potts chain, it is much more natural to take h and J fields from independent distinct quasi-periodic potentials. The above analysis for the Fibonacci fixed point will not apply for such initial distribution. The system, however, still flows to sequences, but the fixed point oscillates between multiple sequences. Though the details of these sequences differ, we observe numerically that they have same universal features which leads to same spin and correlation length exponent as in above calculations (see main text). We remark that our argument only used the probabilities/ratio of various sub-patterns and letters, which could be same for a wide class of sequences. The details of the sequence is unimportant. As an example, for many choices of global phase we get a 3 letter sequence defined by the inflation rules $A_1 \to A_1BA_2BA_1$, $A_2 \to A_2BA_1BA_2$, $B \to A_1BA_2$. This sequence has same ratios and probabilities of relevant sub-patterns as the Fibonacci sequence and hence the above calculations carry forward.

Supplementary Note 2.

A. Symmetric MBL RG ($\beta^I = 1$)

1. Flow to Fibonacci Sequence

The arguments above can be readily generalized to the symmetric MBL RG (see main text). The RG assumes alternating thermal and insulating blocks parametrized by "lengths", $\ell^{T/I}$. The RG flow is dictated by the decimation of the lowest length to get a new length given by the rule,

$$\ell^{T/I}_{\text{new}} = \ell^{T/I}_{n-1} + \beta^{I/T}\ell^{I/T}_{n} + \ell^{T/I}_{n+1}, \tag{6}$$

where $\beta^I = 1/\beta^T \geq 1$. We take the initial distribution of lengths to be a quasi-periodic potential given by $\ell^{T/I}_n = W^{T/I}(1+\cos(2\pi\varphi n+\theta))$. The initial length distribution can be thought of as a sequence like in the case of XXX

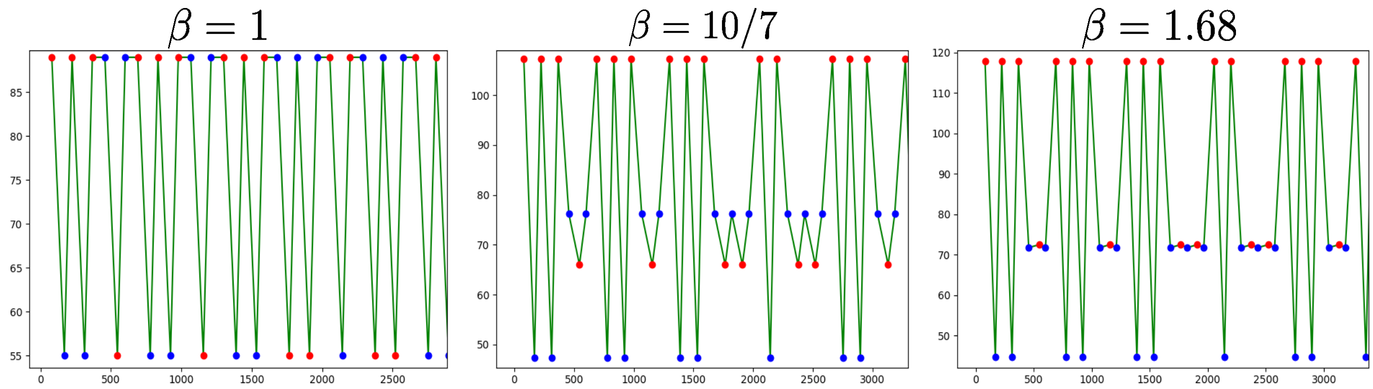

Supplementary Figure 2. Critical self-similar sequence for the MBL transition RG showing the lengths ℓ_n after 3 Fibonacci step for different values of β^I, and $\delta W = 0$. The red (blue) dots represent the thermal (insulating) blocks. We see that on increasing β^I slightly, the length of thermal blocks become greater but the the sequence structure is not broken. However, when β^I is increased further, low thermal blocks become greater than high insulating blocks (see $\beta^I = 1.68$).

spin chain, and for the symmetric case of $\beta^I = 1$ we get a Fibonacci sequence under RG with the following values,

$$\begin{aligned} A_m(n) &= \sum_{i=-\frac{F_{3m+2-1}}{2}}^{i=\frac{F_{3m+2-1}}{2}} (1+\cos(2\pi\varphi(\mathrm{n}-\mathrm{i}))) \\ &= F_{3m+2} + \cos(2\pi\varphi \mathrm{n})\left(1 + 2\frac{\cos\left(\pi\varphi\frac{\mathrm{F_{3m+2}+1}}{2}\right)\sin\left(\pi\varphi\frac{\mathrm{F_{3m+2}-1}}{2}\right)}{\sin(\pi\varphi)}\right) \\ &= F_{3m+2} + \left(1 + \frac{\sin(\mathrm{F_{3m+3}}\pi\varphi) - \sin(\pi\varphi)}{\sin(\pi\varphi)}\right) \times \cos(2\pi\varphi\mathrm{n}) \\ &= F_{3m+2} + \frac{\sin(\mathrm{F_{3m+3}}\pi\varphi)}{\sin(\pi\varphi)}\cos(2\pi\varphi\mathrm{n}) \\ &\xrightarrow{m\to\infty} F_{3m+2}, \end{aligned} \tag{7}$$

since $\sin(F_{3m+3}\pi\varphi) = 0$ as $F_{3m+3}\varphi \approx F_{3m+4}$. Similarly one can show that,

$$\begin{aligned} B_m(n) &= F_{3m+1} + \frac{\sin(\mathrm{F_{3m+1}}\pi\varphi)}{\sin(\pi\varphi)}\cos(2\pi\varphi\mathrm{n}) \\ &\xrightarrow{m\to\infty} F_{3m+1} \end{aligned} \tag{8}$$

Notice that $A_m - B_m = F_{3m+2} - F_{3m+1} \approx \varphi^{3m+1}(\varphi - 1)$, implying that under RG the initial length distribution flows to a fixed point characterized by infinite difference in length. Thus the assumption that $\ell_n \ll \ell_{n\pm1}$ gets better with the RG flow.

As we increase β^I slightly, the thermal blocks are "favored" by the RG rules. This changes (7) and (8) slightly to give (ignoring fluctuations),

$$\begin{aligned} A_m^T &= F_{3m+2} + \delta_{1,m}^T, \\ A_m^I &= F_{3m+2} - \delta_{1,m}^I, \\ B_m^T &= F_{3m+1} + \delta_{2,m}^T, \\ B_m^I &= F_{3m+1} - \delta_{2,m}^I, \end{aligned}$$

where $\delta_{1,2}^{T,I} > 0$, and represents the fact that the size of insulating blocks are reduced due to $\beta^T < 1$ and the size of

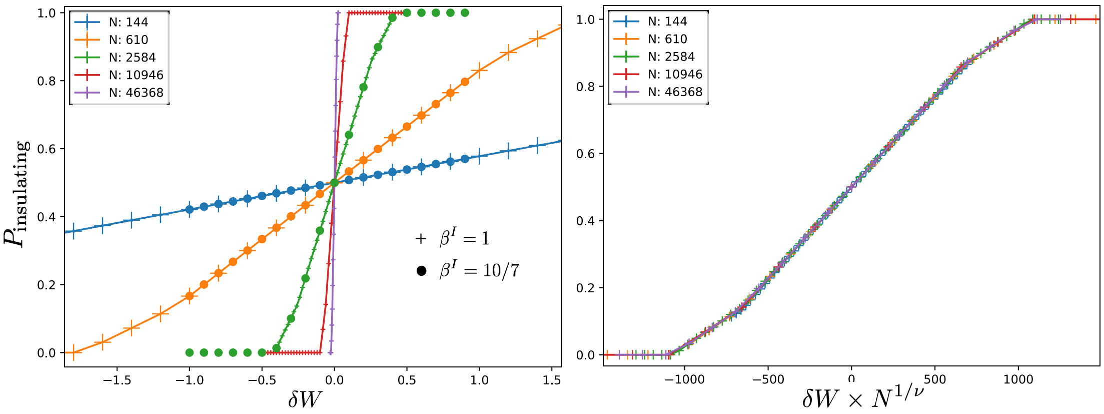

Supplementary Figure 3. *Left:* Probability of getting an insulating block at the end of RG is plotted against $\delta W \equiv W^I - W^T$ for N initial blocks. The '+' markers correspond to $\beta_T = \beta^I = 1$ (symmetric case), while the 'o' markers correspond to $\beta^I = 10/7$. The data for both β^I overlap, as expected from the arguments given in the text. *Right:* Finite size collapse for $\beta^I = 1$ with $\nu = 1$. The error bars represent standard error.

thermal blocks are increased due to $\beta^I > 1$. Under RG we will have,

$$\begin{aligned}
A^T_{m+1} &= 3A^T_m + 2\beta^I B^I_m = F_{3(m+1)+2} + 3\delta^T_{1,m} - 2(\beta^I - 1)\delta^I_{2,m}, \\
A^I_{m+1} &= 3A^I_m + 2\beta_T B^T_m = F_{3(m+1)+2} - 3\delta^I_{1,m} - 2(1 - \beta_T)\delta^T_{2,m}, \\
B^T_m &= 2A^T_m + \beta^I B^I_m = F_{3(m+1)+1} + 2\delta^T_{1,m} - (\beta^I - 1)\delta^I_{2,m}, \\
B^I_m &= 2A^I_m + \beta_T B^T_m = F_{3(m+1)+1} - 2\delta^I_{1,m} - (1 - \beta_T)\delta^T_{2,m}.
\end{aligned}$$

The first term in the above expression increases by a factor of φ^3 and the correction due to the asymmetry between thermal and insulating blocks also increases by an almost same factor. Thus if initially the corrections are small enough we see that the sequence structure is not broken under the RG flow. But for a large asymmetry, $\delta_{1,2}$ can be large enough to destroy the sequence pattern (as we get $B^T > A^I$). In fact studying the dependence of $\delta^{T,I}_{1,2}$ on β^I, we find that for $1 < \beta^I < 1.6$ we have the same Fibonacci self-similar sequence as in symmetric case. This can be confirmed numerically by running the RG for different values of β^I. In the left pannel of Fig. 3 we compare transition data for $\beta^I = 1$ and $\beta^I = 10/7$. They are overlapping perfectly in agreement with our argument.

2. *Correlation length exponent*

We now compute the critical exponent ν in the symmetric case ($\beta^I = 1$). The mechanism responsible for driving the system to a phase (either thermal or MBL) is by introducing an asymmetry in the initial distribution via $W^I \neq W^T$. This leads to formation of "defects", see Fig. 4.

Let us assume that $W^T = 1$ and $\delta \equiv (W^I - 1) > 0$ is small, so that the change in insulating blocks close to the red dash line in Fig. 4 can be written as, $W^I(1 + \cos(2\pi\varphi n + \theta)) \equiv 1 + \cos(2\pi(\varphi n + \Delta) + \theta)$, where Δ is a function of δW with $\Delta \propto \delta W$ for small δW (provided the derivative of the initial potential is well defined at that point), see Fig. 4 The phase plays no important role so we take $\theta = 0$ for simplicity. At criticality ($\delta W = 0$) and at the boundary of the minima region, i.e on the dashed red line in Fig. 4, two adjacent insulating and thermal blocks have the same length, that is, if ℓ_n lies on the red line then $\{n\varphi\} = 1 - \{\varphi\}/2$ and $\{(n+1)\varphi\} = \{\varphi\}/2$. Now, imagine a local minimum insulating block slightly below the dashed line, i.e $\{n\varphi\} = 1 - \{\varphi\}/2 - \gamma$, then the adjacent thermal block will be above the red line by same amount with $\{(n+1)\varphi\} = \{\varphi\}/2 - \gamma$. As we move away from criticality $\delta W \neq 0$, we have

$$\{n\varphi\} \xrightarrow{\delta W \neq 0} 1 - \{\varphi\}/2 - \gamma + \Delta.$$

If $-\gamma + \Delta > \gamma$ then the insulating block will become larger than the neighboring thermal block. This implies that any insulating block for which

$$1 - \{\varphi\}/2 - \Delta/2 < \{n\varphi\} < 1 - \{\varphi\}/2$$

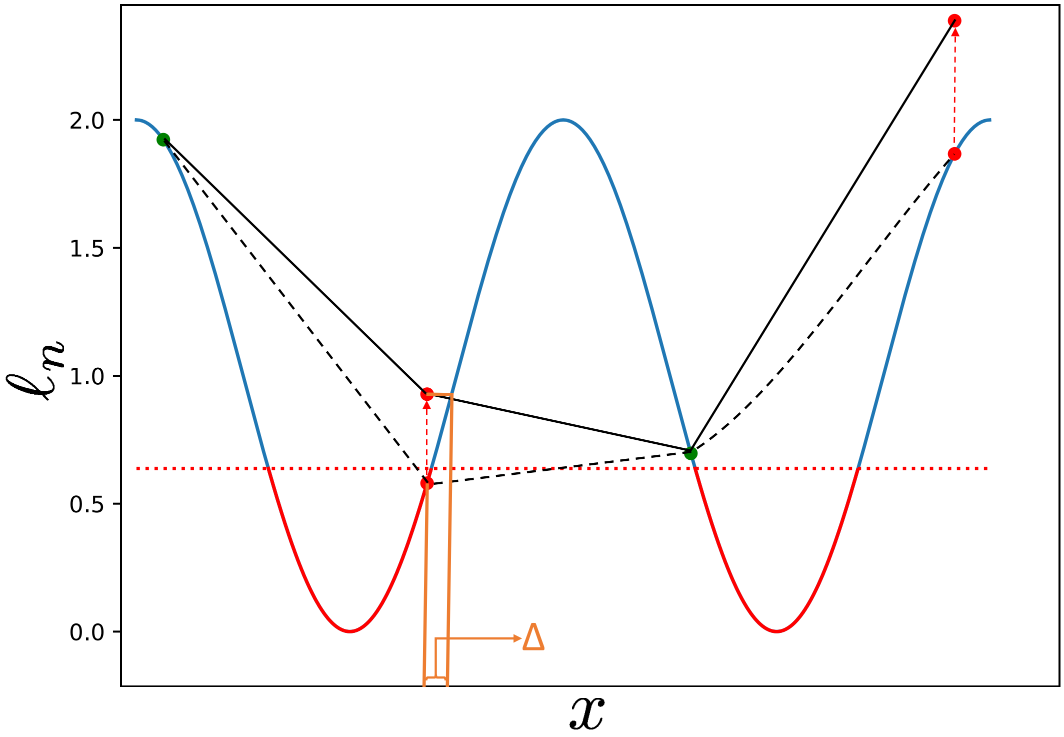

Supplementary Figure 4. Formation of a defect in the sequence. The green dots represent thermal blocks and red dots are insulating. The red dashed line represents the boundary for a block to be a local minimum: all the local minima are below that line for $W^T = W^I = 1$. Here 4 consecutive blocks are shown. As we increase W^I, the thermal (red) dots go up. The initial potential (dashed black line) gets transformed into black line, showing the shift of the minima from the red dot to the green dot.

leads to a defect.

To compute the correlation length, suppose that the block at n_0 is arbitrarily close to the red line and satisfies the above criterion for creating a defect. We are now interested in the position of the next defect in the sequence pattern. Let n_0+k be the position of the next defect. We want k to satisfy $-\Delta/2 < k\varphi - l < 0$ for some integer l. Since n_0+k is the first defect after n_0, we should also impose the constraint that $0 < p\varphi - q < 1/2$ or $-1/2 < p\varphi - q < -\Delta/2$, $\forall p < k$ and for some $q \in \mathbf{Z}$. We know by Diophantine approximation that the quantity $|a\varphi - b|$, where a, b are integers, is lowest among all the integers $c < a$ *if and only if* a is a Fibonacci number. Thus k has to be a Fibonacci number, $k = F_{i_0}$ with $k\varphi - F_{i_0+1} = (-\varphi)^{-i_0}$ and the constraint that $\varphi^{-i_0} < \Delta/2 < \varphi^{-(i_0-1)}$. (If $k\varphi - F_{i_0+1} > 0$, we can simply take the next Fibonacci number with $k = F_{i_0+1}$.) The distance between defects sets the correlation length $\xi = F_i \approx \varphi^i \sim \Delta^{-1} \sim \delta W^{-1}$, which implies that $\nu = 1$. We checked numerically that $\nu = 1$ leads to a perfect finite size collapse of the probability for the RG to end with an insulating block (Fig. 3).

B. Singular example:

The above argument for $\nu = 1$ suggests that more singular potentials can lead different exponents $\nu > 1$. When we wrote $\Delta \propto \delta W$, we implicitly assumed that the derivative of the potential is not singular at the point where $\{\varphi x + \theta/(2\pi)\} = \frac{\{\varphi\}}{2}$. However there exist some functions for which this is not true. For example, consider $\ell_n = f(\cos(2\pi\varphi n + \theta))$ with $f(x) = 2 + \mathrm{sgn}\left(\cos(2\pi\frac{\{\varphi\}}{2}) - \mathrm{x}\right)\sqrt{|\cos(2\pi\frac{\{\varphi\}}{2}) - \mathrm{x}|}$. $f(x)$ is a bounded monotonic function in the range $(-1, 1)$ and hence flows to sequence under RG, but it has a singular derivative at $x = \cos 2\pi\frac{\{\varphi\}}{2}$. This implies that $\delta W \sim \sqrt{\Delta}$, so we expect $\nu = 2$ in this case, as confirmed by numerical simulations of the RG (Fig. 5).

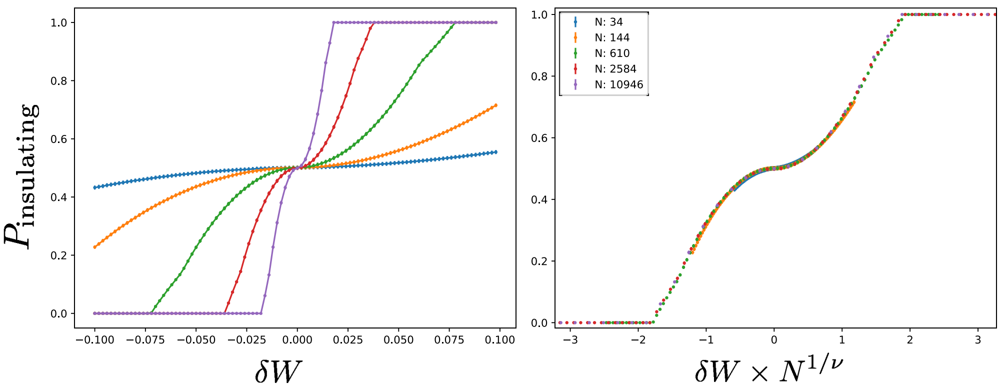

Supplementary Figure 5. Transition and scaling collapse for the symmetric) MBL transition RG ($\beta^I = 1$) for a singular initial distribution $\ell_n = 2 + \text{sgn}\left(\cos(2\pi\frac{\{\varphi\}}{2}) - \cos(2\pi\varphi\text{n} + \theta)\right)\sqrt{|\cos(2\pi\frac{\{\varphi\}}{2}) - \cos(2\pi\varphi\text{n} + \theta)|}$. The collapse gives $\nu = 2$. The error bars represent standard error.

C. $\beta^I > 1$

As mentioned above, slightly increasing β^I is an irrelevant perturbation to the sequence, and does not alter the transition values. But if β^I is increased beyond a certain threshold, the asymmetric perturbation can dominate the sequence values and break down the sequence pattern (see Fig. 2). We get different self-similar sequences as β^I is increased. The next self-similar sequence is for $3 < \beta^I < 12.75$, see Fig. 6. This sequence is self-similar under one Fibonacci step. More concretely if the sequence size is $F_{3(m+1)}$ then under RG, the sequence pattern will re-emerge when the system size becomes F_{3m}. To formalize this, we define the so-called substitution matrix of the sequence. If we define $w_1 = A^T,\ w_2 = A^I,\ w_3 = B^I,\ w_4 = B^T$, then the substitution matrix M is given by $M_{ij} = \#w_i$ in the inflation rule for w_j.

For the Fibonacci sequence discussed above for the case of β^I close to 1, the inflation rules were $A^T \rightarrow A^T B^I A^T B^I A^T$, $B^T \rightarrow A^T B^I A^T$, and so on. This corresponds to the substitution matrix,

$$M = \begin{bmatrix} 3 & 0 & 0 & 2 \\ 0 & 3 & 2 & 0 \\ 2 & 0 & 0 & 1 \\ 0 & 2 & 1 & 0 \end{bmatrix}. \tag{9}$$

Let $w = [\#w_1, \#w_2, \#w_3, \#w_4]$ be the vector representing the number of various letters in the initial word sequence. Let λ_i be the eigenvalues of M (with λ_1 the largest value) and v_i being the corresponding eigenvectors. We assume that we can write $w = \sum_i a_i v_i$ with some real coefficients a_i. The vector Mw denotes the number of letters after a single application of inflation rules. Thus after n inflations, the total number of letters is given by,

$$\begin{aligned} N_n &= \sum_{i,j} (M^n)_{ij} w_j \\ &= \sum_{i,k} (\lambda_k^n a_k (v_k)_i \\ &= \lambda_1^n a_1 \sum v_1 \left(1 + \left(\frac{\lambda_2}{\lambda_1}\right)^n \frac{a_2 \sum v_2}{a_1 \sum v_1} + ...\right) \\ &\approx \lambda_1^n a_1 \sum v_1, \end{aligned}$$

implying that the number of letters changes by a factor of λ_1 under a single application of the inflation rule. For (9) $\lambda_1 = \varphi^3$, implying that under one Fibonacci step, the number of blocks is decreased by a factor of φ^3.

Moving back to the sequence in Fig. 6 for $3 < \beta^I < 12.75$, the substitution matrix is given by

$$M_2 = \begin{bmatrix} 2 & 0 & 0 & 1 \\ 0 & 4 & 3 & 0 \\ 1 & 0 & 0 & 0 \\ 0 & 3 & 2 & 0 \end{bmatrix}, \tag{10}$$

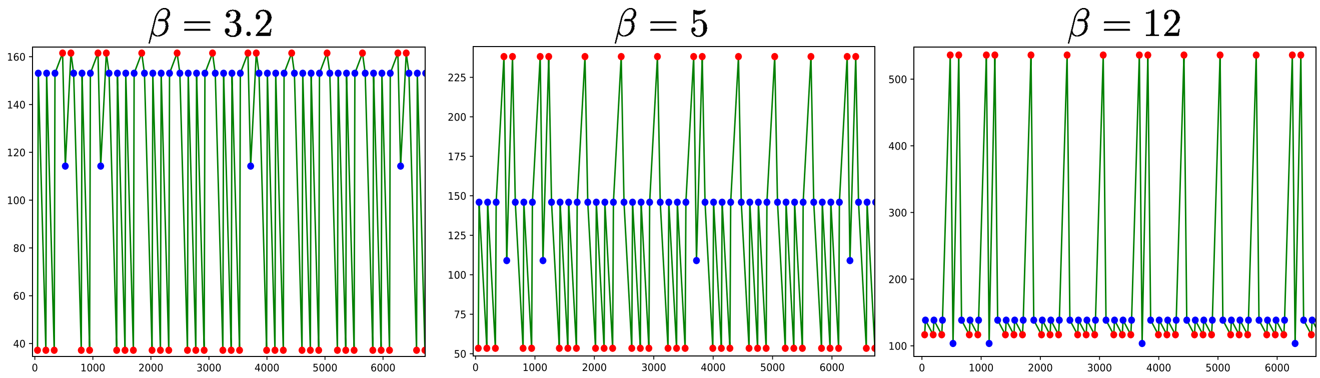

Supplementary Figure 6. Critical self-similar sequence after 3 Fibonacci step for different values of β^I at the critical value of $\delta W \approx 1.97$. Red dot represent the thermal blocks and blue represents insulating. We see that on increasing β^I, the length of thermal blocks become greater and length of insulating blocks smaller (relative to one another). We checked that the sequence structure is preserved at all Fibonacci steps. Eventually on increasing β^I further the sequence pattern will get destroyed, i.e the smaller thermal blocks will be larger than the largest insulating block.

with the largest eigenvalue given by φ^3. This shows that the sequence repeats itself after a single Fibonacci step. We remark that the range of β^I over which the above sequence is well defined can be identified semi-analytically. We can obtain the lengths of the block after the 1st Fibonacci step as a function of β^I by summing over the cosines (like we did for symmetric case). Then the lengths at further steps can be calculated as follows,

$$\begin{bmatrix} A^T_{m+1}(\beta^I) \\ A^I_{m+1}(\beta^I) \\ B^I_{m+1}(\beta^I) \\ B^T_{m+1}(\beta^I) \end{bmatrix} = \begin{bmatrix} 2 & 0 & \beta^I & 0 \\ 0 & 4 & 0 & 3\beta_T \\ 0 & 3 & 0 & 2\beta_T \\ 1 & 0 & 0 & 0 \end{bmatrix}^m \begin{bmatrix} A^T_1(\beta^I) \\ A^I_1(\beta^I) \\ B^I_1(\beta^I) \\ B^T_1(\beta^I) \end{bmatrix}. \tag{11}$$

Eq (11) can be calculated numerically and we can check that the sequence pattern, $A^T_m > A^I_m, B^T_m, A^I_m$ and $A^I_m > B^I_m$, is true at all Fibonacci steps for $3 < \beta^I < 12.75$.

As we increase β^I further, we get different sequences with different periods of self-similarity. For $62 < \beta^I < 78$, we have another sequence whose substitution matrix is given by,

$$M_3 = \begin{bmatrix} 2 & 3 & 3 & 1 \\ 0 & 4 & 3 & 0 \\ 1 & 17 & 14 & 0 \\ 0 & 16 & 13 & 0 \end{bmatrix}. \tag{12}$$

The largest eigenvalue of M_3 is φ^6. This implies that the sequence repeats itself after two Fibonacci steps, i.e if the system size is $N = F_{3m}$ then under RG flow the sequence re-emerges when the system size is $F_{3(m-2)}$. This can be checked numerically (see Fig. 7). A nice finite size collapse for the transition is observed only for system sizes of the form $F_{3\times(2m+1)}$, in contrast to the previous two transitions, for which collapses were obtained for system sizes of the form F_{3m}.

We have also observed more complicated sequences for larger values of β^I, with a period of 3 Fibonacci steps. Studying such sequences numerically is however very challenging as extremely large systems are needed. We expect that as one increases β^I, the transition is controlled by larger and larger sequences with complicated inflation rules and scaling of the form φ^{3k}. Our defect argument for $\nu = 1$ is however quite general, and applies generally to all these self-similar sequences.

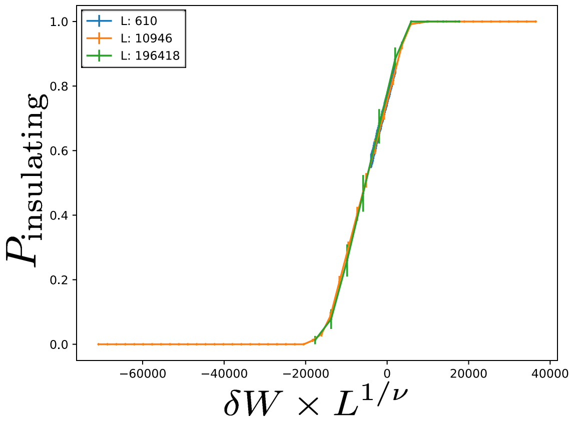

Supplementary Figure 7. Collapse for $\beta^I = 70$ with $\nu = 1$. For this value of β^I, the critical sequence repeats itself after two Fibonacci steps. The error bars represent standard error. Unlike Fig. 3, in which we had a crossing between systems with lengths of the form F_{3m}, in this case the crossing is seen only for system sizes $F_{3\times 5} = 610,\ F_{3\times 7} = 10946$ and $F_{3\times 9} = 196418$, i.e for system sizes of the form $F_{3\times(2m+1)}$.
